# Supplementary material for: Resynthesis: Marker-Based Partial Reconstruction of Elite Genotypes in Clonally-Reproducing Plant Species
Source: Front Plant Sci. 2020 Aug 7;11:1205. doi: 10.3389/fpls.2020.01205 (PMC7427350; doi:10.3389/fpls.2020.01205)
Supplement: Supplementary file 1 [file DataSheet_1.pdf]

Table S1. Description of the peach SNP markers included in the Fluidigm chip used for the construction of the 'Sweet dream' (SD) map

SNP name	Abbreviation (Map)	Chromosome	Physical position (bp)	SNP type	SD map	cM[1]
SNP_Pp01-8080	na[2]	Pp01	8080	A/C	monomorph	um[3]
SNP_IGA_2651	na	Pp01	883576	T/C	No call	um
SNP_IGA_17419	SI17419	Pp01	6000280	T/C	yes	0.0
SNP_IGA_19514	SI19514	Pp01	6872054	T/C	yes	4.5
SNP_IGA_31646	na	Pp01	12187249	T/C	monomorph	um
SNP_Pp01-15435423	na	Pp01	15435423	A/G	monomorph	um
SNP_IGA_67137	SI67137	Pp01	20219128	T/G	yes	25.4
SNP_IGA_79455	SI79455	Pp01	23529157	T/C	yes	30.1
SNP_IGA_86968	SI86968	Pp01	26640558	T/G	yes	36
SNP_IGA_103771	na	Pp01	33744976	A/G	monomorph	um
SNP_IGA_120784	na	Pp01	41413142	T/C	No call	um
SNP_IGA_125219	na	Pp01	46984218	T/C	no	um
SNP_IGA_123719	na	Pp01	47532400	A/G	No call	um
SNP_IGA_238135	na	Pp02	241474	T/C	No call	um
SNP_IGA_136625	SI136625	Pp02	2002076	A/G	yes	0.0
SNP_IGA_148760	SI148760	Pp02	3109681	A/G	yes	3.3
SNP_IGA_195134	SI195134	Pp02	6545323	A/G	yes	9.6
SNP_IGA_218801	SI218801	Pp02	9053696	T/C	yes	13.1
SNP_IGA_242118	SI242118	Pp02	13717350	A/G	yes	17.7
SNP_IGA_252957	SI252957	Pp02	15849694	A/G	yes	23.6
SNP_IGA_258854	SI258854	Pp02	17219077	A/G	yes	26.8
SNP_IGA_266063	SI266063	Pp02	19744284	T/G	yes	34.3
SNP_IGA_275502	SI275502	Pp02	22301505	T/C	yes	41.1
SNP_IGA_283325	SI283325	Pp02	25849495	T/C	yes	52.5
SNP_IGA_285058	SI285058	Pp02	26895362	A/G	yes	58.8
SNP_IGA_898654	SI898654	Pp03	742389	A/G	yes	0.0
SNP_IGA_299796	na	Pp03	4209014	A/C	monomorph	um
SNP_Pp03-3917979	na	Pp03	3917979	T/C	No call	um
SNP_IGA_322698	na	Pp03	9911321	A/G	monomorph	um
SNP_IGA_428056	na	Pp03	11876389	T/C	monomorph	um
SNP_IGA_894039	na	Pp03	13664157	T/C	No call	um
SNP_IGA_325850	SI325850	Pp03	15520683	A/C	yes	27.6
SNP_IGA_344612	SI344612	Pp03	19098840	A/G	yes	35.9
SNP_IGA_349233	SI349233	Pp03	20300608	T/C	yes	43.9
SNP_IGA_356179	SI356179	Pp03	22294942	A/G	yes	53.4

[1]cM= genetic distance in centimorgans
[2]na=not assigned
[3]um=unmapped

Table S1 (continued)

SNP name	Abbreviation (Map)	Chromosome	Physical position (bp)	SNP type	SD map	cM
SNP_IGA_368926	SI368926	Pp04	407324	T/C	yes	0.0
SNP_IGA_379856	SI379856	Pp04	1477791	T/C	yes	3.5
SNP_IGA_389204	SI389204	Pp04	4843247	A/G	yes	15
SNP_IGA_395253	SI395253	Pp04	6202308	T/C	yes	21.1
SNP_IGA_398213	SI398213	Pp04	6895567	A/G	yes	25.5
SNP_IGA_403741	SI403741	Pp04	9106604	A/G	yes	33.2
SNP_IGA_409453	SI409453	Pp04	10396616	T/C	yes	37.7
SNP_IGA_420819	na	Pp04	13949755	A/C	monomorph	um
SNP_Pp04-13754607	na	Pp04	13754607	A/G	monomorph	um
SNP_IGA_440116	SI440116	Pp04	16084694	T/G	yes	52
SNP_IGA_454568	SI454568	Pp04	18760831	A/G	yes	57.3
SNP_IGA_480804	SI480804	Pp04	20068418	A/G	yes	58.2
SNP_IGA_513502	SI513502	Pp04	22616919	A/G	yes	59.5
SNP_IGA_528949	SI528949	Pp04	24187959	A/G	yes	63.7
SNP_IGA_544495	SI544495	Pp05	610569	T/G	yes	0.0
SNP_IGA_548037	SI548037	Pp05	1376475	T/C	yes	4.1
SNP_IGA_553456	SI553456	Pp05	2477309	A/G	yes	8.2
SNP_IGA_571548	SI571548	Pp05	5485331	A/G	yes	17.4
SNP_IGA_572589	SI572589	Pp05	5813029	A/G	yes	18.6
SNP_IGA_585810	SI585810	Pp05	9347092	T/C	yes	22.3
SNP_IGA_591439	SI591439	Pp05	11196070	A/G	yes	34.7
SNP_IGA_595126	SI595126	Pp05	12682824	T/C	yes	42.9
SNP_IGA_596393	SI596393	Pp05	13411012	T/C	yes	45.9
SNP_IGA_600493	SI600493	Pp05	14879113	A/C	yes	53.5
SNP_IGA_602605	SI602605	Pp05	16636368	T/C	yes	58.9
SNP_IGA_616458	na	Pp06	1304512	A/G	No call	um
SNP_Pp06-2176846	na	Pp06	2176846	T/C	monomorph	um
SNP_IGA_610889	na	Pp06	3034364	T/C	No call	um
SNP_IGA_627328	na	Pp06	7550350	T/G	monomorph	um
SNP_Pp06-7216093	na	Pp06	7216093	A/C	monomorph	um
SNP_IGA_639062	na	Pp06	11085127	A/C	monomorph	um
SNP_IGA_640395	SI640395	Pp06	11298491	T/C	yes	0.0
SNP_IGA_655825	SI655825	Pp06	14681941	T/C	yes	5.4
SNP_IGA_663057	SI663057	Pp06	17959563	A/C	yes	11
SNP_IGA_671806	SI671806	Pp06	21011215	T/C	yes, dominant	18.5
SNP_IGA_682254	SI682254	Pp06	24511760	T/C	yes, dominant	29.7
SNP_IGA_691624	SI691624	Pp06	27207318	A/C	yes	42.8
SNP_IGA_696341	SI696341	Pp06	28500652	T/C	yes	46.4
SNP_IGA_700653	SI700653	Pp06	30012439	A/C	yes	52
SNP_IGA_726222	na	Pp07	350629	A/G	No call	um
SNP_IGA_717591	na	Pp07	2354039	T/C	No call	um
SNP_IGA_704075	na	Pp07	4239920	A/C	No call	um

Table S1 (continued)

SNP name	Abbreviation (Map)	Chromosome	Physical position (bp)	SNP type	SD map	cM
SNP_Pp07-5647370	S0756473	Pp07	5827566	A/G	yes	0.0
SNP_IGA_752104	SI752104	Pp07	8182487	T/C	yes	4.5
SNP_IGA_758767	SI758767	Pp07	10388929	A/C	yes	12
SNP_IGA_774557	SI774557	Pp07	13833942	A/G	yes	21.7
SNP_IGA_776826	SI776826	Pp07	14891881	A/G	yes	27
SNP_IGA_781003	SI781003	Pp07	16422167	A/G	yes	38.4
SNP_IGA_784777	SI784777	Pp07	18244540	A/G	yes	46.2
SNP_IGA_794167	na	Pp08	373237	A/G	Not clear	um
SNP_IGA_797492	na	Pp08	1248336	T/G	monomorph	um
SNP_IGA_803699	na	Pp08	2564296	A/G	Not clear	um
SNP_Pp08-3702593	na	Pp08	3702593	T/C	monomorph	um
SNP_IGA_821894	SI821894	Pp08	5071328	T/C	yes	0.0
SNP_IGA_851849	SI851849	Pp08	10999017	T/C	yes	11.1
SNP_IGA_860815	na	Pp08	13540876	A/G	monomorph	um
SNP_Pp08-13670362	na	Pp08	13670362	T/C	No call	um
SNP_IGA_871082	SI871082	Pp08	16740766	A/G	yes	28.3
SNP_IGA_878044	SI878044	Pp08	18644875	A/G	yes	38.6
SNP_IGA_883524	na	Pp08	20833164	T/G	Not clear	um
SNP_IGA_884755	SI884755	Pp08	21455147	A/G	yes	53.5

Table S2. Sequences of the KASPar assay primers and chromosomal region where the SNPs used in this paper

SNP name	A1	A2	C1	Sequence
SNP_Pp01-8080	GAAGGTGACCAAGTTCATGCTGTGAAAACACCGTATCTTAACAGTAAGTT	GAAGGTCGGAGTCAACGGATTGAAAACACCGTATCTTAACAGTAAGTG	ATGTTGCGTTCTCAGATATATAGGCCTTT	CTTACCGGTAAGATATGATGTTTTTAGAAAAACAAGTTATTTCCTCTATCAATTACGAGTAAGATGTTGCGTTCTCAGATATATAGGCCTTTTACACTAT[A/C]ACTTACTGTTAAGATACGGTGTTTTCACAAAAATAAGTTATGTAGTCCACGAATTACGGGTAAGACGTGGGGTTCTCATATATCCACGGTTTTACACTAT
SNP_IGA_2651	GAAGGTGACCAAGTTCATGCTCACTCACCACCATGTTGTTATTATTTG	GAAGGTCGGAGTCAACGGATTCCACTCACCACCATGTTGTTATTATTTA	GGGAAAGTGATGGAAGCATTTTGTTTGAA	CATTGAGTATCTCAATTGGTAGTTGCAGATCTTCTTCAACTGCTGAGGGCTCTCATGGGGAAAGTGATGGAAGCATTTTGTTTGAAGAACAAAGAGAAGC[C/T]AAATAATAACAACATGGTGGTGAGTGGAAGAGGAAGCAGTAAAATCAGAGGAGAAGTAGTGTTGATGAAGAAGAATGTAATGGACTTCACTGATATCAGA
SNP_IGA_17419	GAAGGTGACCAAGTTCATGCTGTGCAATGCCCAACAGGTGTC	GAAGGTCGGAGTCAACGGATTCGTGCAATGCCCAACAGGTGTT	GTGATCATTGTGGTGAAAAGAAACATACTA	GCGACAATAGCCACATGAGCTTTATCGATCAGTTGTTCCTTTTTTAAACGCCTCTATTCCTTCTCCTAATCTAGTACCTCGTGCAATGCCCAACAGGTGT[C/T]GATAGTATGTTTCTTTTCACCACAATGATCACACTTTAGTTTGCTTTTTTACAGAAGTCATGCGACAAGGTAGAACGGTAGAAGAGCCAAACCCTATAGG
SNP_IGA_19514	GAAGGTGACCAAGTTCATGCTCATTTTCTGGGGTAGTGGCACC	GAAGGTCGGAGTCAACGGATTAACATTTTCTGGGGTAGTGGCACT	CTGCCTGAGCCGGCGTCCA	CAAGGTATGCACAATGGTATGCAAACCAAAGGCGATACGACTGCGCTTTGAGGCACTCAAACGGCCCTTATGGAGAGAACATTTTCTGGGGTAGTGGCAC[C/T]GGGTGGACGCCGGCTCAGGCAGTGGCGGCATGGGCTTCGGAGAGCAGGTGGTACAATTACTGGTCTAATTCTTGTGCTAGAGGGCAGGAATGTGGGCATT
SNP_IGA_31646	GAAGGTGACCAAGTTCATGCTATTTGACAACAAGAGTGAGTCTGGC	GAAGGTCGGAGTCAACGGATTATATTTGACAACAAGAGTGAGTCTGGT	CATCCGAATTAGCTTCTGGTGGTGA	TTGTTATATCAATTGATGTTTAAAGTACGCAAATTTGATTGATATGTAGTTGATTATTCTGTAAACAGGTGGTGATATTTGACAACAAGAGTGAGTCTGG[C/T]TCACCACCAGAAGCTAATTCGGATGAGGATGAAAGTGGTGCTGCTTTTCTTGTACGAATCTTGAGGTACCTTGAGACACCTCAATATTTGAGAAAAGCTC
SNP_Pp01-15435423	GAAGGTGACCAAGTTCATGCTAACTCGCACAAACGGAAGGATAGA	GAAGGTCGGAGTCAACGGATTCTCGCACAAACGGAAGGATAGG	TAACATGAAATTCCTCATCTTTGCATGGTA	GACAAAGGACTTGGAAGAACGAGAAGAAGGAGACGCACCAAACATTGGCCATAAACAACAAGCCATCCAACAATTACAACTCGCACAAACGGAAGGATAG[A/G]GAGACATATCCGCCACTACCATGCAAAGATGAGGAATTTCATGTTATCCTTGACACGATGATTGCTGATGGTGCAATCAGGCCTGTTAGACCCCACAAAA

SNP_IGA_67137	GAAGGTGACCAAGTTCATGCTACACAAAGTAAAAAACTTACCGATCCG	GAAGGTCGGAGTCAACGGATTATAACACAAAGTAAAAAACTTACCGATCCT	GGATCTTATCCATGAAAATAGGAGGGAA	AAAATAATTTCTTCTTTTCTTTTCTGTTTTTTTTTTTCAGAAATATAAAGTTCCCTGTTGTTCTGTTACAAATAACACAAAGTAAAAAACTTACCGATCC[G/T]GGGATGTAGAGTCGGCGCTTCCCTCCTATTTTCATGGATAAGATCCCTTCATCAAGTCCCTTGATCACCTGTTCTACTCATTCAGATTAACAATTATTAA
SNP_IGA_79455	GAAGGTGACCAAGTTCATGCTAAGCGCGTTACCATACCCGAC	GAAGGTCGGAGTCAACGGATTCAAGCGCGTTACCATACCCGAT	GGCGGACCTCGGGACGCTT	CTGGAGCCGAACACTCTAGAGACCAGGCCCAGCTCCTTAGCCTCCGAACTCGAAAAGGTCCGACCCGTAAGCGCTAATTCAAGCGCGTTACCATACCCGA[C/T]GATGCTGGGTAGCCTCTGAAGCGTCCCGAGGTCCGCCGTGATCGCCAAGTCCACCTCTTTCACCGAAAAGAACGCGTCCTTGCTGCAATACCTTATGTCA
SNP_IGA_86968	GAAGGTGACCAAGTTCATGCTCATAATGCTGAAGTGCAAACAGGC	GAAGGTCGGAGTCAACGGATTCCATAATGCTGAAGTGCAAACAGGA	CAGATGTAGAAGTTCCAATTGCATGCAT	TAAAATAACATGGCTACTTTGCATTTATACTTCCTCCTCAAAGTTTGATAAATTTTTTTTGCAGATGTAGAAGTTCCAATTGCATGCATCATTCTGATAG[G/T]CCTGTTTGCACTTCAGCATTATGGAACTCATAGGGTTGGGTTCTTGTTTGCTCCTATAGTTCTGACATGGCTTCTGTGCATTAGTGCAATTGGTCTTTAT
SNP_IGA_103771	GAAGGTGACCAAGTTCATGCTTTCTTGAATAGCTGCATCTCTTTCA	GAAGGTCGGAGTCAACGGATTTCTTGAATAGCTGCATCTCTTTCG	GCAACATCAGCCATCAATGAAACAGATAA	TTGCGGCATCTCGCTGCAGGAATGCCATGTCTCGCTCTTGAAGGGCAGCTTTTTTCTCAGAAAATGCCAAATTTCTTTCTTGAATAGCTGCATCTCTTTC[A/G]CCCATTATGGCCATTATCTGTTTCATTGATGGCTGATGTTGCATCAACCACTAAAACAAACAGAGAGATACAAAATTTCATCAGATATGCCTCTCAAGTG
SNP_IGA_120784	GAAGGTGACCAAGTTCATGCTACCCTCATCACTATGGTTGTTATCG	GAAGGTCGGAGTCAACGGATTACCCTCATCACTATGGTTGTTATCA	GACAATGATGAAGAAGAGAATGAGGTTGAA	CCAAAATACCAGTTGATAGTGATTCTGAAGATTCTGAAGATACAGCTGATGACAATGATGAAGAAGAGAATGAGGTTGAAGACGAGGACAATGGTAATAG[C/T]GATAACAACCATAGTGATGAGGGTAAGTGTTAAGTTCATAAATATGTCATGCGCTACCTATAATAAATGACAGCTAGGGCTATGAGGATGAGAATTTTCT
SNP_IGA_125219	GAAGGTGACCAAGTTCATGCTAACTTGCACATTTGAGGCATGAAGC	GAAGGTCGGAGTCAACGGATTCAACTTGCACATTTGAGGCATGAAGT	GGTCATGAAGGTGGAATTTTTACTGTTGAA	AAGACAAAGAACAAAACACCAACAGTCGTTGAAACATAATGTTAACAATGCATGAATACCCTGTAACTGGATCAACAACTTGCACATTTGAGGCATGAAG[C/T]GGGGCTTCAACAGTAAAAATTCCACCTTCATGACCTTGGCCTTGCTTGATATGTTTCTTTACCTGCAAAGACAGAGTACTTGTTCAACATTGGAATAGCC
SNP_IGA_123719	GAAGGTGACCAAGTTCATGCTAAGCAACTGTGGATTGGATGGACT	GAAGGTCGGAGTCAACGGATTGCAACTGTGGATTGGATGGACC	TTTCAGGTTTTTGTGGCTGCAACACTAAA	GTTTGTCGAATATGAGATTGTGTAGATGCATGTAACAAATCTCTTTTGTTATTATCCTTTTCAGGTTTTTGTGGCTGCAACACTAAAGAGAAAGCAGGTT[A/G]GTCCATCCAATCCACAGTTGCTTCATGGAAGCCAAAACAATGTGCCAAATGACACAACCACTTTCTCTACTGACGCTGGCAGTTTTCTTCACACTGAAAA

SNP_IGA_238135	GAAGGTGACCAAGTTCATGCTCCGATTCAACATGACTAATACGGG	GAAGGTCGGAGTCAACGGATTCCCGATTCAACATGACTAATACGGA	AAAGGCATGTTCCGCAATTTGATCACTT	TTCATCGTCTCATCAATCACCTGCCGCGCATGGTAGTAAGAGCAATTCAAAAGGCATGTTCCGCAATTTGATCACTTGTGGTGCTGTCGACACAAATGAC[C/T]CCGTATTAGTCATGTTGAATCGGGCCAATAAAACAACTTCTTCCGACAAGTCTACCAGATGGAGCAGCCAAGACATTATCAAGGCTGATCCTATTTGTAA
SNP_IGA_136625	GAAGGTGACCAAGTTCATGCTGAAATTGTCAAATGTGGATGTAATGCAA	GAAGGTCGGAGTCAACGGATTGAAATTGTCAAATGTGGATGTAATGCAG	CGGATTTTCATTACTATACTGCAAAAGTTT	GTGCGAAAATGATATACGACGCTGATGATCGTGGCGACGTGATTGATGGTGATCTTGGTGAGCACTTTGATCTGAAATTGTCAAATGTGGATGTAATGCA[A/G]GAGAAACTTTTGCAGTATAGTAATGAAAATCCGAATAGAACAGTTGTGAATCCATACATTCATTTCGGGCAACGGTCAATTTGGCCTAGGGGATTGCCTT
SNP_IGA_148760	GAAGGTGACCAAGTTCATGCTGAGAACCAAATTATCTGCCTGTGGTT	GAAGGTCGGAGTCAACGGATTAGAACCAAATTATCTGCCTGTGGTC	CATCTGATGCCTGAGGTCAACCTTT	CGGCCTGTTGGAGTGCAAAGTCAAGCATCCATTCCTCTGCATTTTTCTTTTCGTCCATCATCTGATGCCTGAGGTCAACCTTTTCTGCTTCAGGATCAGG[A/G]ACCACAGGCAGATAATTTGGTTCTCTTGGATTGTACTTCCTTTGTTCCTCCTCATCCACGCTAAGTCTCTTGAATTTGCTTCCTCTTCGGTAGTTGCAGG
SNP_IGA_195134	GAAGGTGACCAAGTTCATGCTCCAGATGAGATACCCTTGAATGCAA	GAAGGTCGGAGTCAACGGATTCAGATGAGATACCCTTGAATGCAG	CCGGATAGAACTGCATTACAGTTTATGAA	TTGGTTTCTTAATATTATTATCTTTTCATCTCAGGGAACAAGAAAAATTAAAGGCATCATGGTGAAGTTTCCCAAGCCAGATGAGATACCCTTGAATGCA[A/G]AAAGCTTCTTCGGGATGGTAAATCTTGAAATTTTCATAAACTGTAATGCAGTTCTATCCGGATATGTTGAATATCTACCCAACGAGTTGAGGTTCATTGA
SNP_IGA_218801	GAAGGTGACCAAGTTCATGCTGGTCTTCAGTTTCCAGGTTGCG	GAAGGTCGGAGTCAACGGATTTGGTCTTCAGTTTCCAGGTTGCA	AACTCCACTGAAAGATTTTGGATGTGCAT	AGAGGAAGCCTTACCAACTACTGATGGAAACCTTTTGACAGCGGGCATACGAAGGCAAACTCCACTGAAAGATTTTGGATGTGCATCAGTGGGTTTCAAG[C/T]GCAACCTGGAAACTGAAGACCAAAGGAAAAAAAAAAAAACAATTATCATATGAATATGAGCTGAATGAGATGCAACATTGGCAAATTACGAAACAGGAGC
SNP_IGA_242118	GAAGGTGACCAAGTTCATGCTATAGCCAATGCGATGAACACCAGT	GAAGGTCGGAGTCAACGGATTAGCCAATGCGATGAACACCAGC	CTAACAACATCTTGGCCGTCGGATT	TCAGATGGGGTGATCTGCTACTGGACCCAGATCCTAACAACATCTTGGCCGTCGGATTGACGGGGCTGCTGACGTGGGCAAGCGTGCAGGTGCTATGGCA[A/G]CTGGTGTTCATCGCATTGGCTATACTTGTTGCTGCTGTTAAGTATTCTTTCATAGCTGCTGTTCTTCTTTTCATTCTCATTGCCCTTCTTTAAAAATTCT
SNP_IGA_252957	GAAGGTGACCAAGTTCATGCTCCAATAATCCTCCACGTAAGCAACA	GAAGGTCGGAGTCAACGGATTCAATAATCCTCCACGTAAGCAACG	CCTATAACGACGTGCTTTCTTAGGATAAA	AATTCTTCATTACGTAATGTAATTGAGCGATGTTTTGGTGTGTTGAAAGCTCATTTTTCAATTTTGAAATTGATGCCCAATAATCCTCCACGTAAGCAAC[A/G]AAGTATCCCTTGCAAGTTGTGTGTTACATAATTTTATCCTAAGAAAGCACGTCGTTATAGGTTGTTTGAAGAGTTTCAAGTAGAAGACATGATTGTTGAG

SNP_IGA_258854	GAAGGTGACCAAGTTCATGCTTACACAAGGGGTCTTTGAGCAGTT	GAAGGTCGGAGTCAACGGATTCACAAGGGGTCTTTGAGCAGTC	TCCTATAGGTACTTCCTGTGATGATTGAT	TATGGAGAATTATTTGGAAAATTCCATATTGTTCTTAACCCGTTATGCTCCATCCTATAGGTACTTCCTGTGATGATTGATGTTGGAACTAACAATGAAA[A/G]ACTGCTCAAAGACCCCTTGTGTAAGTGTTTCCTCATCTAATCTTCAATACGCGGTACAAAAAACAGCTATTTTGATTATTTACAATGTTAATATGATGCT
SNP_IGA_266063	GAAGGTGACCAAGTTCATGCTATCATTTGGAGAGTAACCAAATATGATTG	GAAGGTCGGAGTCAACGGATTCATCATTTGGAGAGTAACCAAATATGATTT	AAATGCCAATTCGATTTCACTCAACACTTT	TGTAGTTGGGTTATGGCTCAGAAAATCCTCACATAGTTTTGGGTGAGGAGTTTGGCTACACCATGTTTTCACATCATTTGGAGAGTAACCAAATATGATT[G/T]GAGAGAGATAAAGCACCAAGAAAAGTGTTGAGTGAAATCGAATTGGCATTTTTGGATGGCTTTTGATCAAGAAATGTGTATGAAATATGGGCGATTTGGG
SNP_IGA_275502	GAAGGTGACCAAGTTCATGCTGAGAGCCAGGGAAATTGCCAAG	GAAGGTCGGAGTCAACGGATTGGAGAGCCAGGGAAATTGCCAAA	GCAGCTTAAGAGAAACATCTCAATACCTT	CCACATGCCATACCTCCATTACTAATAAACAGAAGCCAAGAATGACTGAAACATATGCAGCTTAAGAGAAACATCTCAATACCTTGCGTTTAGCAATATC[C/T]TTGGCAATTTCCCTGGCTCTCCACTCATCAGCTTCTCGATCAGCTTCTTCGAATCTTTCGCGCTCCTGTGCATCACCAAAGCAATAAAAAATGTAAGCAA
SNP_IGA_283325	GAAGGTGACCAAGTTCATGCTACAGATTATATGAAGCAGCTTCTTTCG	GAAGGTCGGAGTCAACGGATTCACAGATTATATGAAGCAGCTTCTTTCA	GTGATGCAAGTGAAACCAACCAAATTCTA	TTAAATACACATAATCAGAAATCTTACCCAAGTGGTCTTCATTCAAGTGATGCAAGTGAAACCAACCAAATTCTACTTACATGTGATGAGGGTATTTGAG[C/T]GAAAGAAGCTGCTTCATATAATCTGTGATGTGGTACCCACCGATGTTAGTTCGGCAGCTTCCTTGATACATAGGTTCGCCGTCAATAAACTGGGAATGAA
SNP_IGA_285058	GAAGGTGACCAAGTTCATGCTCACCACTACATTGAAGGAATAGTATGTT	GAAGGTCGGAGTCAACGGATTACCACTACATTGAAGGAATAGTATGTC	GGCTTGGCTTGTCAGCTGAGCT	TGATTGCTCCCATACGCCAATTCAACATCAATGTGCAAACAAATTCCCCATGTACAGTGGGAATACTTGTTGACCGAGGCTTGGCTTGTCAGCTGAGCTC[A/G]ACATACTATTCCTTCAATGTAGTGGTGATTTTCATTGGTGGAGCAGACGACCGTGAGGCGTTGGCCTACGCAGCACGGATGTCTGGCAATCCGGACGTGG
SNP_IGA_898654	GAAGGTGACCAAGTTCATGCTGGTCAGACAATTGAAGAGGCACA	GAAGGTCGGAGTCAACGGATTGGTCAGACAATTGAAGAGGCACG	TCTTTCACTTGTAACAGAGCTTGTGCAT	AGTTGGGCACTAATGGCGGCGAAAGAAGCAATTTTTGTTGAAGCTGCAAATGGATTTGACTTGCAACTGGTAGCACCTGGTCAGACAATTGAAGAGGCAC[A/G]GAGTGGAATCAAAGGGCATGCACAAGCTCTGTTACAAGTGAAAGAGCTGATAGATTTAGAGTCATGGAGAGAAGTACAAATAGCTCTCAGGAAGAGCTCA
SNP_IGA_299796	GAAGGTGACCAAGTTCATGCTGCCACTGGAGAAAGCCTAACCA	GAAGGTCGGAGTCAACGGATTCCACTGGAGAAAGCCTAACCC	CACATATTGCTGCTAAAGTAGCTGCTAAA	GTAGCCAAACCTACAAAAGCCAATTGAAGAATGTTAACTTTTAGGCCTATTCAGTCACAAGAAATCTTACAAGAGCAGAGCCACTGGAGAAAGCCTAACC[A/C]AGTTCATATGATTTAGCAGCTACTTTAGCAGCAATATGTGCCGAAATCTTTCTAATGTTTGTAAATGGAGGGTATATGAGTCCCTTGTCATAGTCTTCCT

SNP_Pp03-3917979	GAAGGTGACCAA GTTCATGCTGAA TCTAGCCAAGAG GCTTGCTG	GAAGGTCGGAGTC AACGGATTATGAA TCTAGCCAAGAGG CTTGCTA	GGAGCTTGCTCT ATCTGACTGCAA	TCAAAAGTGGATGGAAGTGAACTAGCAGATGAGACATTGTATA GGCAAATGGTGGGGAGCTTGCTCTATCTGACTGCAACCAGACCA GATATCATGTTTG[C/T]AGCAAGCCTCTTGGCTAGATTCATGCAT AATCCAACCAAGAAGCACATGGGAACAGCAAAAAGAGTGCTGA GATATGTTCAAGGCACCATAAACTATGGA
SNP_IGA_322698	GAAGGTGACCAA GTTCATGCTAGA CGGTAATGGTTG CATGAGCAA	GAAGGTCGGAGTC AACGGATTGACGG TAATGGTTGCATG AGCAG	TCATAGGACTCA ATAAGTCCTATA GCGTA	GGCTCGGTAGATGCATGAGACGTTCCAAATTCTCGTTGCTTCTCT TCTTGAGAAATCAAAGAATATGCCTTGCGAACAGACGGTAATGG TTGCATGAGCA[A/G]AATTTGTCCTTGCACTACGCTATAGGACTT ATTGAGTCCTATGAGGAATTCCATAAGTGCATTTCTCTCCTCTTG TTCGCTATGTTTCTTCATAGCACCA
SNP_IGA_428056	GAAGGTGACCAA GTTCATGCTGTA CGCATCCTATGT TTGGACCC	GAAGGTCGGAGTC AACGGATTGTACG CATCCTATGTTTG GACCT	GACCTTGTCATA AACAAAAGGAA GACCAT	TTGTTGATGTCCTTTCGGTAAAAGAATTTCCAAGAAATCTGTTAC TTCAAAATTTGCCGCTGGATTGTGATATACTCTGTACGCATCCTA TGTTTGGACC[C/T]GAGAGTGGAAAAAATGGGTGGAATGGTCTT CCTTTTGTTTATGACAAGGTCAGGGTCGGAGGTGACGAATCAAG AGTGTCACGGTGCAACAAATTTCTGG
SNP_IGA_894039	GAAGGTGACCAA GTTCATGCTCCT CTTATTGTGGTG CTCCC	GAAGGTCGGAGTC AACGGATTAACTC CTCTTATTGTGGTG CTCCT	CTAAACATTTGC GAACAGATAAAG ACGCTA	TGAACTCATAAGAGAATCATTACATTCCTTCCCAGACTTGGGCC ATCCGGGCCCATGGACTAGTGGTCCCGAAACTGAACTCCTCTTA TTGTGGTGCTCC[C/T]ATAAATCTTTTCTAGCGTCTTTATCTGTTC GCAAATGTTTAGTTGGATCAACCAGAAAACCAGAAACAGCTTCC TCCCTGTGAGGGTTGAACATTTCACT
SNP_IGA_325850	GAAGGTGACCAA GTTCATGCTTAC GCATTGTGCGGA AATAATGCAAA	GAAGGTCGGAGTC AACGGATTACGCA TTGTGCGGAAATA ATGCAAC	CAATACTCCGAG CCTGTGGACATA T	GAACTTGACCTGACCTCCCTATGATATCCACCATACATGAGTAA TGCTCCAATTGAGGGTGTAAACCATAGTCATTACGCATTGTGCG GAAATAATGCAA[A/C]CCCTTTTCAACTTGTCCTATATGTCCACA GGCTCGGAGTATTGAAACAAAAGTGGAGTGGTTTGGCTTAACAT TCTCAAGCTGCATGTTCTCAAAAATTC
SNP_IGA_344612	GAAGGTGACCAA GTTCATGCTTCC ATCATGAAATAT CACTGGCTTT	GAAGGTCGGAGTC AACGGATTCCATC ATGAAATATCACT GGCTTC	ACAGCAAGAGTG GTTCAAATTCAG TGAT	AATTAATTATGTGTATTGGATTGTAACAGGAAGCCTATGTAGTG AACAAGGAAGACAGCAAGAGTGGTTCAAATTCAGTGATGCCAA GGCAAAAATACCC[A/G]AAGCCAGTGATATTTCATGATGGAAGG CTAGCATTCTTGCCTACACCATCTGCAACTCTTGCCATGTTCATG TGGCTCCCTCTTGGAATAATCCTAGCCA
SNP_IGA_349233	GAAGGTGACCAA GTTCATGCTCCG CCATCTTCGCTG TCCG	GAAGGTCGGAGTC AACGGATTATACC GCCATCTTCGCTG TCCA	CGTTTCTTTCCAT TCCCGAGCGAAT	GAGGCCCAGGCGCTCGCCAGCAAGCGTCGGCGTATCAGTATCGC ACCCTCTCCAAACTCCGCCGTTTCTTTCCATTCCCGAGCGAATCA CAAAGAAGGCT[C/T]GGACAGCGAAGATGGCGGTATTTACACCG CGGCTCTCAAAGGAAGCCAAAGAGCAGCCCCCGAGCCTCCACG CGGTGGATTTTCGACGGGGAGAGTCAAT

SNP_IGA_356179	GAAGGTGACCAAGTTCATGCTCAGTAGTCAGGATAAATGCATGTGTT	GAAGGTCGGAGTCAACGGATTCAGTAGTCAGGATAAATGCATGTGTC	TTTCATGCGAGGCTCAGAAACAAATGAA	CACTGCCTGCAGAATATGACCGAGCAGACCTTGACGTATACCTCTCCATTCCCCGAACTTTCATGCGAGGCTCAGAAACAAATGAACCATGGTCTTTAAC[A/G]ACACATGCATTTATCCTGACTACTGGAAGCATAGTTCCTTTTACTCCTTTTATGACAGTAGTCTTAATTTGAGTCGTTCTGCTCACACCACTACAATCAC
SNP_IGA_368926	GAAGGTGACCAAGTTCATGCTAGCCCGTTATTCACACCTCTATTC	GAAGGTCGGAGTCAACGGATTGAGCCCGTTATTCACACCTCTATTT	GTTGGCATTGCAAAGAGGGCATGAT	TATAGCTCTGATTCTCCTCATCTCTTTCTTTAAATAAGTTGCTGACCAGTATATACCAAGTCAAATGATTTGGAACGAGCCCGTTATTCACACCTCTATT[C/T]AAAAGTAAACAAGCATCATAAATCATGCCCTCTTTGCAATGCCAACTGATCAAAGTGTTATAAGTAATAACATCAGGCCACATCCCTTCAACTTGTAACC
SNP_IGA_379856	GAAGGTGACCAAGTTCATGCTGTTTGCTTCGATCTGGTCATCC	GAAGGTCGGAGTCAACGGATTGTGTTTGCTTCGATCTGGTCATCT	AATGAACCCAAAAATATCAGATTTCGGCAT	ATTTTGTAAATCAGTTGTGCATGTTTCGGATTGAGGGCAGAGAGTGAGAGTTTTACTATGTGCCAACAACTCTGTTTGTGTTTGCTTCGATCTGGTCATC[C/T]CCGAACATTCTTGCCATGCCGAAATCTGATATTTTTGGGTTCATTGAGGCATCCAGTAAAACATTGCTTGCTTTCAAATCCCTATGGATAATTTTTAGTC
SNP_IGA_389204	GAAGGTGACCAAGTTCATGCTAGAGTCTGAAATTCCCTTCCCTCAT	GAAGGTCGGAGTCAACGGATTAGTCTGAAATTCCCTTCCCTCAC	CATGGGGATCTTGCCATGTAGTCAA	AATTGTAAAGCTTCCTAATCCAATCCATGTGCTTTGCCTCATCTTCATGGGGATCTTGCCATGTAGTCAAGTACTGAATTTTAAAGAGAATTCCATTTCT[A/G]TGAGGGAAGGGAATTTCAGACTCTGAAATTCTGCTCATCTGTCCTCCATATGGATTGAAAATCACCAGAGGGCTCTCTTCCTCCATCAGCCTTTTCCAAA
SNP_IGA_395253	GAAGGTGACCAAGTTCATGCTCAGACATCATCCTCTTCACG	GAAGGTCGGAGTCAACGGATTGCTCAGACATCATCCTCTTCACA	GTAGGCCAAGGATCAAGCACATTCAA	CGTTAGGATGTACAGAATTAACGAAATAAATCAATGTTAACTTGTGGCTGCTTCATTGTAGGCCAAGGATCAAGCACATTCAAAACCTAGTTCAAGAAAA[C/T]GTGAAGAGGATGATGTCTGAGCAATTACAGATTCCTTCCACTGCCTCTGGTCGTCAATTAAAACCAAATTCTGATAATCGCTTCACCAAACCAAGCTTTA
SNP_IGA_398213	GAAGGTGACCAAGTTCATGCTTCCTTTCCAAAATCTTAGCTCTTGTA	GAAGGTCGGAGTCAACGGATTCCTTTCCAAAATCTTAGCTCTTGTG	ATGGGTTGAACTCAAGTTCTGAGAAAGTT	AATCTCCATGATGATGAAGATCAGCAGAAGCACCATCAACACCTACACTAGCACGCTTAAAGCTCTTGGACTTCTTCCTTTCCAAAATCTTAGCTCTTGT[A/G]GTACCTCCTGGGGTCACAACTTTCTCAGAACTTGAGTTCAACCCATTAGGATCATTTCCTCTCTTTATTGCTGGTGGCCTTGGAGACTTTGACTTCGGAG
SNP_IGA_403741	GAAGGTGACCAAGTTCATGCTAAAGACCTGGCTTTTGAGTTGCCT	GAAGGTCGGAGTCAACGGATTAGACCTGGCTTTTGAGTTGCCC	ACCTCAACTTCAAAACCACCAGTGTTTTA	TCTTGAGACTTGGGAAACACCCTGACGCAGGAGGAGCATTGGTAATACAGTTTGACCTCAACTTCAAAACCACCAGTGTTTTACACATGAAAATGAATTG[A/G]GGCAACTCAAAAGCCAGGTCTTTATCTGTTGTTTCAAAACAAAGATCAAGTTCAACAACATTATGCTGGATGGCAGTTCGAATCCAACCATCAATACCAG

SNP_IGA_409453	GAAGGTGACCAAGTTCATGCTCCGATATCTACAGCTTTGGAGTC	GAAGGTCGGAGTCAACGGATTGTCCGATATCTACAGCTTTGGAGTT	ATGGGTTTCCTCCCGCAAGCAATTT	CACAAACAACAAATGTTGCTGGAACCAGGGGTTATATGGCTCTGGAATACGTTACCACAGGAAAGGCTAGCATGGAGTCCGATATCTACAGCTTTGGAGT[C/T]GTTGCTTTGGAAATTGCTTGCGGGAGGAAACCCATTGATCTCAGTTTAGAAAATAGCCAAATCGAAATGGTGGAGTGGGTGTGGGAGCTTTATGGAGAAG
SNP_IGA_420819	GAAGGTGACCAAGTTCATGCTGTAGACGACACCATTGACGACCT	GAAGGTCGGAGTCAACGGATTAGACGACACCATTGACGACCG	CGACGTATATGGGTTTGGATTTGATTCTT	AAGAAACTACCAGAAATACCAACAGAATATCTAATCGATGACCTCTACTACTTTTGCGACGTATATGGGTTTGGATTTGATTCTTCAACTCATCAACACA[A/C]GGTCGTCAATGGTGTCGTCTACAGAACAAGTATCCGTGATGATGATGAAGGAGGGGTTCAGTTTAATGTGTATACATTGGAAACTAATTCTTGGCGACAG
SNP_Pp04-13754607	GAAGGTGACCAAGTTCATGCTCAACGTGTTGTATTTGATTGAATACCATT	GAAGGTCGGAGTCAACGGATTCAACGTGTTGTATTTGATTGAATACCATC	GACTATAAGGTTTAGCGCTCTCGTCTT	CGAATTTCATGCCAAAAGTTTATATGTATTTTTTTAAGCTTATGAATTTAAGACTATAAGGTTTAGCGCTCTCGTCTTTTCATATAAATATTATTGTTTA[A/G]ATGGTATTCAATCAAATACAACACGTTGCTATAAGGATTATGATTTTGTTTTTTCATAAAAATTAAAATTGCTTTGGTTGGAGTATGACCAAACTAGAAG
SNP_IGA_440116	GAAGGTGACCAAGTTCATGCTGAACATTTCTATACTGCCACGACC	GAAGGTCGGAGTCAACGGATTCGAACATTTCTATACTGCCACGACA	CTGTGCAATGAAGTGCAGCAACACTT	AATGAAGGAAAGAACATGTGGTGCATGGTCCAGGACTTTGTCCTTGGGGGCATGCGTGTACTGTGCAATGAAGTGCAGCAACACTTTTGAGCGCACCATC[G/T]GTCGTGGCAGTATAGAAATGTTCGGCCTTAACACTCTTATCTCATCATCTCATGCTCAATAAGATCATCATTCTCTCTTTATTAGTGGATATCCAATCGT
SNP_IGA_454568	GAAGGTGACCAAGTTCATGCTGTCCTCTTCTTCTCCTTCTCCTTT	GAAGGTCGGAGTCAACGGATTCCTCTTCTTCTCCTTCTCCTTC	GAGTGGAAGAAGAGGAAAGAACATTTCTA	TCCATGTCCAGTCCAGTGAGCGACACAAAAAGCGGCGCCTAAGAGCAGAGTCAGAGTGGAAGAAGAGGAAAGAACATTTCTATAGCAGATGCGGCAAATG[A/G]AAGGAGAAGGAGAAGAAGAGGACATAGTTTGCTTAGATGAATCTTTCTTCATCGATGACAAGTAAGAGGAAGATACAGTCTTTTTAAGTTTTGTGGTTAT
SNP_IGA_480804	GAAGGTGACCAAGTTCATGCTACCATTTACATCACCTATTGTGTTCGA	GAAGGTCGGAGTCAACGGATTCCATTTACATCACCTATTGTGTTCGG	TCAATCAGACAACTGGGTTCCGGTA	TAGATGCATATGTTGTGAATGTTCCTAGCATGATGAGCATAGATTTCAAGAATCCCTGAAAAGTGATCAGAATCACCATTTACATCACCTATTGTGTTCG[A/G]ATTCAGGTTGTACCGGAACCCAGTTGTCTGATTGAATGATTCCATCAGATCCGAATATAGCTTCTTCCCCTTGAGGATATTCTAATATTTTCTGTTTGGT
SNP_IGA_513502	GAAGGTGACCAAGTTCATGCTAGTATCTTCAAGTTAATAGGCCTGGT	GAAGGTCGGAGTCAACGGATTGTATCTTCAAGTTAATAGGCCTGGC	TTAGTCCATCGTTGGACCTGGTCTT	TGGTGATGGCATTCCATGCACCGTCCACGTTGATTTTGAGATGCCTCATGGGTGGTTTAGTCCATCGTTGGACCTGGTCTTGGTTGGAACAAGGTGTCCC[A/G]CCAGGCCTATTAACTTGAAGATACTCAAGTAGTCTATTATGTGCAATATGGCCTACCAGGCTTGGGCATGAACTTTTATTGTTCCATGGTAAACCATTTC

SNP_IGA_528949	GAAGGTGACCAAGTTCATGCTGTCACATATCTCCCACTCCCACT	GAAGGTCGGAGTCAACGGATTCACATATCTCCCACTCCCACC	CATCAAAACCTAGGAAACTTTGTTGGGAA	GCGCATAGGTGGTCCAGGATGAGGCATTAAAACAGATTGAATCTAGCCTCTGTGCAATCCATCAAAACCTAGGAAACTTTGTTGGGAAACAAAAGCAGAT[A/G]GTGGGAGTGGGAGATATGTGACAGAACTTTCGCAACCAATCGTATTTTTGGTTGGAATGGCAGTGAGGTCAGCTGAGCCGTAAAGAACATTATTATTACC
SNP_IGA_544495	GAAGGTGACCAAGTTCATGCTTCTCCACCACTTCCTCTACC	GAAGGTCGGAGTCAACGGATTACTTCTCCACCACTTCCTCTACA	GCCGGAGAGAGATACATATGCACAT	GGACGCTCGTGGATGTTCGTGATTTAGCAGAATCACTGCTTCTTGTATACAACAAGTCTGAGGCCGGAGAGAGATACATATGCACATCGCACGAGATCGG[G/T]GTAGAGGAAGTGGTGGAGAAGTACTTGAGGCCTGCATATCCTAACTACAACTATCCCAACAAGTAAGTTTTTCTTACCCTCCAATAATTAGTTTAGTTGG
SNP_IGA_548037	GAAGGTGACCAAGTTCATGCTTTTCATTCGATAGTGCCCTGC	GAAGGTCGGAGTCAACGGATTCCTTTTCATTCGATAGTGCCCTGT	CAAGCCTCTAACACTTCCAACCTGAT	TGGCAGCCATCGGAATACGAGTATTACAGTGGATAACGTATCATGGCTTAGCGTTGAATGTCACCACTGACTTAACCCCTTTTCATTCGATAGTGCCCTG[C/T]GGGTTACGAGACTATCAGGTTGGAAGTGTTAGAGGCTTGCTGAAGGAATTTCAGTCATCCACTGACTGCGAAAGAGCACGTCTACCTGATCCTGATGATG
SNP_IGA_553456	GAAGGTGACCAAGTTCATGCTAAGTTCATATTCTACTTGGCTGAGGA	GAAGGTCGGAGTCAACGGATTAGTTCATATTCTACTTGGCTGAGGG	GAGTTTGATTATGTTTGCGAATTGCCGTT	ATGCAACTATTTCAATAAGTTACCTAGAGAAGTAATATTGGCTTCCCCAATATCAAGCTCTTCGGTTTTCATCCTAAGTTCATATTCTACTTGGCTGAGG[A/G]TTGCTTTCCAGAACGGCAATTCGCAAACATAATCAAACTCTATCTTCTTCTCGATTTCAGATTCTAACTGTTTGAATTTGTTGTCATATGACTGCAACTG
SNP_IGA_571548	GAAGGTGACCAAGTTCATGCTAAGGTCAATTCCAGCACTCTTGGTT	GAAGGTCGGAGTCAACGGATTGGTCAATTCCAGCACTCTTGGTC	TGCAAATTGCTCACCAGAAGGGCTA	GGGTTGTATTAAACTTTACCTGCATGGAAATGAAAGACAGAGAACAGCCTGCACATGCAAATTGCTCACCAGAAGGGCTAGTGCGGCAGGTAAAAATGGC[A/G]ACCAAGAGTGCTGGAATTGACCTTGCAGGAGAGAATGCATTGGAGAGGTATGATACTGGTGCATTTGAACAAGTTTTGGCAACAAGTAGATCAGATTCCG
SNP_IGA_572589	GAAGGTGACCAAGTTCATGCTGTTTACCACTGGGATCTGAGAACA	GAAGGTCGGAGTCAACGGATTTACCACTGGGATCTGAGAACG	AAAGCGGTACCATTTATGCAACCTTCAT	AGATGAATGGAACTGTTCGCTCTTTATCTTTTGCCAATGATGGGAAGCAATTATTGAGCTCTGGTGGTGATGGACAGGTTTACCACTGGGATCTGAGAAC[A/G]GGGGCCTGCTTCCACAAGGCACTTGATGAAGGTTGCATAAATGGTACCGCTTTGTGTACATCTCCAAATGGGACCATGTTTGCTGCTGGTTCAGACAGTG
SNP_IGA_585810	GAAGGTGACCAAGTTCATGCTCAGTGCAGCTGCTTCAGATTCTG	GAAGGTCGGAGTCAACGGATTCAGTGCAGCTGCTTCAGATTCTA	GCAGAAGCATGCAGATACGAAGAATTTAT	GTTTTATCCGGAAAACTACAACTCAGTAGTTCAATGCGATTGGCTGCAGAAGCATGCAGATACGAAGAATTTATCGATACCAACCTGAAAGGGAAATTCT[C/T]AGAATCTGAAGCAGCTGCACTGGCAAAAATCGCGCTTGTATGCACCCACGAGCTTCCCGACCACAGGCCAACAATGCAGGAAGTAATTCTGGAGCTGAGT

SNP_IGA_591439	GAAGGTGACCAAGTTCATGCTGATGTGAGCATGATTGGGCAA	GAAGGTCGGAGTCAACGGATTCTGATGTGAGCATGATTGGGCAG	CACAATCTCCCTCTGCAGAGTAGAA	TGTTAGATTTAGTAAACAACTTGGGGACAATTGCAAGGTCTGGGACAAAGGAGTTTATGGAGGCATTGCAGGCAGGGGCTGATGTGAGCATGATTGGGCA[A/G]TTTGGTGTGGGTTTCTACTCTGCAGAGGGAGATTGTGACCACAAAGCACAATGATGATGAGCAGTATGTGTGGGAGTCTCAGGCTGGTGGTTCTTTCACC
SNP_IGA_595126	GAAGGTGACCAAGTTCATGCTGAGACCCAAAAACTGCCGGTAAG	GAAGGTCGGAGTCAACGGATTAGAGACCCAAAAACTGCCGGTAAA	GTTCGGAGACTTGAAAATTCCGCGAT	ATATCTTCTCGAACCGGTCAAGTGGGTCCTCCTTGTTCTTCACGCCGAAAACGCCGTCGTTCGGAGACTTGAAAATTCCGCGATTGGGGTTGGAGTTTTC[C/T]TTACCGGCAGTTTTTGGGTCTCTAAAGTTGAAGGCTTTGGGCGGGGGGAGCGGCGAGAATCGGAAGCTATCGGCGTCGAGGCCGGCCATGAGCTCCCATG
SNP_IGA_596393	GAAGGTGACCAAGTTCATGCTGGCATAGAGTTCAGCAGAGAGG	GAAGGTCGGAGTCAACGGATTTGGCATAGAGTTCAGCAGAGAGA	GCACTCAACCCTTTAACTGAGGATCTT	CACCGAGAGAGCACATAGAAGAGATAAGGAAGAAAAAGTTCTCCATAGGAGCAGATGCACTCAACCCTTTAACTGAGGATCTTCACCAGGCTATCAAGAC[C/T]CTCTCTGCTGAACTCTATGCCAAAGATGTTCACTTCCTCATGGAACTCATCCAGGTTGTTTATTTTAAATATACATTCCTACATATCATGACTTAAGTTT
SNP_IGA_600493	GAAGGTGACCAAGTTCATGCTAACCAATTGATGCAAGAAAATAGTGAGAAT	GAAGGTCGGAGTCAACGGATTCCAATTGATGCAAGAAAATAGTGAGAAG	CATCCATATTATCCACTTCGTCTTCAGAA	CATCCTGTGTGGTATCACTACCTTCTCCTAGAGTGACCTCATCAGGCTGAATTTCATCCATATTATCCACTTCGTCTTCAGAACTGTCATCAGAATCTGA[A/C]TTCTCACTATTTTCTTGCATCAATTGGTTCACAGAATTGTCATCCTCTATCACTTCCTTAGCAACATCAACATCCATGACTTTAACTTTAAGTCCTGGAA
SNP_IGA_602605	GAAGGTGACCAAGTTCATGCTGGTCTCTATCGAGGTTTTGGGG	GAAGGTCGGAGTCAACGGATTGGGTCTCTATCGAGGTTTTGGGA	CAAAGTACATGCCTCGATACAGTGTAAT	GAGATGCTTACCTCCAAAGACCCAACCAAAACAATAGGCTTCATGGTGTCATAAATCCCAAAGTACATGCCTCGATACAGTGTAATCCCAATAATTGAAA[C/T]CCCAAAACCTCGATAGAGACCCGCTATTCCATCACTTGACAAGGTTTTACTATAAACATCTAGAATCCCTCTGAACTGGCGCTGACCATTAACTGAAGAC
SNP_IGA_616458	GAAGGTGACCAAGTTCATGCTAAGTTGGAATGTCAGAGGTTTGGGT	GAAGGTCGGAGTCAACGGATTGTTGGAATGTCAGAGGTTTGGGC	TCTCTCAATAAATCATTGAGAGCTCTGAAT	CTTTCAATTGCACACTAGTACATTTAGTCTCCATAAGGAAAATTACATGGGGATTTTTCTCTCTCAATAAATCATTGAGAGCTCTGAATGTCCGAGGATT[A/G]CCCAAACCTCTGACATTCCAACTTAAGATATTCAATGCCTTTGTGCCCAACATCTTTGTGTCCACCACGGATTCATACATGACAATCTCAGTATTTTTTC
SNP_Pp06-2176846	GAAGGTGACCAAGTTCATGCTCCATTGTAGCAAACCAGAATAACAATTG	GAAGGTCGGAGTCAACGGATTCCATTGTAGCAAACCAGAATAACAATTA	CATTGTATTGCCTGATTATTGTGGTTGTAT	ATATTGATGTTATATTGCTGTTATATTGTCATTGTATTGCCTGATTATTGTGGTTGTATTGTGTTAATGAAATAATTTTTTTCATAGTCAGAAATGGAGA[C/T]AATTGTTATTCTGGTTTGCTACAATGGAAAATGGGTAACCTCAAAGAAGATATGCAAATACGAAGGGGGTGACTCAAAAGGCTTAATAGTTCCACGAACC

SNP_IGA_610889	GAAGGTGACCAAGTTCATGCTAGCTTGAGGAAGAAAGTTGTATTATGC	GAAGGTCGGAGTCAACGGATTAAGCTTGAGGAAGAAAGTTGTATTATGT	TTCATCATTGTTTTCTGTGAACAAACCGTT	TGATTTATCAGCGCAGGAAAGTGTCAGCAAAGTTGAGGGAAGAAAAGCAGAATCCCAGCGCAGCGAAGAATTTAAGCTTGAGGAAGAAAGTTGTATTATG[C/T]ACAACGGTTTGTTCACAGAAAACAATGATGAAATGGGTGCTTTAACATCGAAGAGCAAGAACAGTGAATTTAAGGAAGCAAAAGATGCAGCTCCAGGACT
SNP_IGA_627328	GAAGGTGACCAAGTTCATGCTGAGGTTTCTAGTAATCTATTTTCTCTTTG	GAAGGTCGGAGTCAACGGATTCTGAGGTTTCTAGTAATCTATTTTCTCTTTT	ACTCATGCTATGTCTTCTTTTGCAACCAA	ACAACTCTTTCATTTATTTTTATTTTTCAGTCTTTCACTACAAAAGAACTCACATGTATTGTGAAGTTCACTGAGGTTTCTAGTAATCTATTTTCTCTTT[G/T]GGGGGCAGTGCATTACACTCGTTGGTTGCAAAAGAAGACATAGCATGAGTATTACAAGTGGACCTTTTTCGCCTTGGATACGGATAAATGACGGCCAACT
SNP_Pp06-7216093	GAAGGTGACCAAGTTCATGCTATATTTCTTATTTAGGGATATAATTGGAAATAA	GAAGGTCGGAGTCAACGGATTATATTTCTTATTTAGGGATATAATTGGAAATAC	TTCCCATCCAAATCCTAATTAATTTGGAAA	TTATGACCAATTTACCACTAGTAACAATTAAAAATACCAAGGCAAACATATATTACACTTTTTAATGCATATTTCTTATTTAGGGATATAATTGGAAATA[A/C]ATTTTGTTTGGTTTTCCAAATTAATTAGGATTTGGATGGGAAATACAATTTACCACTATAGGATTTGGATTGGTGTCGAAAAAAGGGATTTCTATTAACA
SNP_IGA_639062	GAAGGTGACCAAGTTCATGCTGGTCGCGAGTGTTGTTGTCCTT	GAAGGTCGGAGTCAACGGATTGTCGCGAGTGTTGTTGTCCTG	GCACAGCACAAGGCAGAATCATGAA	GAAGTTGCTTGCATTGCAAAAGCAAAAGCAAAGCTAGAGTTTCAGCGAAAGCACAGCACAAGGCAGAATCATGAACCGTCTGAGTTTGGAGCAGGTGCTC[A/C]AGGACAACAACACTCGCGACCCCAACTCCATTTCAAGTCTCAAGCTCAATCACAAGGCTCTTTCGGATGTACGATGTCGTTTTTCTGCTCTCCCCCTATC
SNP_IGA_640395	GAAGGTGACCAAGTTCATGCTAAACTTAAGAAGCGGAGGCCAATG	GAAGGTCGGAGTCAACGGATTGAAACTTAAGAAGCGGAGGCCAATA	TATTCCTCATATCCAGCAGGGCCAT	TGTAGGCAGCTTCCAATATCTTGAGGTTTTGGGTATGTGCTTCTTCTGGGAAGCTGTAATCAATGTATTCCTCATATCCAGCAGGGCCATCTTTGGTTAC[C/T]ATTGGCCTCCGCTTCTTAAGTTTCTTTGGAAGCTTAGACTGGACTAAACTAACATCACCGAGATCTCCGAAGCCAGCCTCCACATTCAACATTTCTTCAA
SNP_IGA_655825	GAAGGTGACCAAGTTCATGCTAGAGGATGTATGTGGTGAAGAGG	GAAGGTCGGAGTCAACGGATTGAGAGGATGTATGTGGTGAAGAGA	TCAAGCGCGCCGTTATCTTGTCAAA	CCACCAGCACAGGGTCGCAGTGGTCGCTGCTAAGCCCATAGCTCAGCTTCTTCAAGCGCGCCGTTATCTTGTCAAAATGCACCGCCTCCTGCCGCCCATC[C/T]CTCTTCACCACATACATCCTCTCTCTCTATCTCTGTCTCTGAACGATTTCGTCGAACAGGTCTTGTGCAAGAGAAAGTGAACAAGGAGGAGACTCTGGGA
SNP_IGA_663057	GAAGGTGACCAAGTTCATGCTAAGGGTCCTACATAATTCTCAAATCCA	GAAGGTCGGAGTCAACGGATTGGGTCCTACATAATTCTCAAATCCC	GAAGACTATCAATGGAGATGACCTTCTTT	AAGGGTCTTCTTCTTGTCTAGTCATGGAGTTCTTCTCCCCCTCAGTCTCCCTATACTTGTTGAGATACCCTTTCAAGGGTCCTACATAATTCTCAAATCC[A/C]AGGGTTGTCATGGCCCAAAGAAGGTCATCTCCATTGATAGTCTTCCTTTTCTCCCTCAGGCACTTGTCAGACGCTTCACCCGTGATGAAGCTTATGAACT

SNP_IGA_671806	GAAGGTGACCAAGTTCATGCTGGGGTTTTGAAGTACTTTACCTATAAG	GAAGGTCGGAGTCAACGGATTATGGGGTTTTGAAGTACTTTACCTATAAA	GGCCGTGCAACTTGGAGCAGTT	GTCAACAACCTTGACAGAAAATGTACATACAATTAACATAACGAAGGTGGCAAATCGCAGGGCCGTGCAACTTGGAGCAGTTTTCTTAATCTTCTTCTCG[C/T]TTATAGGTAAAGTACTTCAAAACCCCATAAGTTTGAATTGCTGTCAAAAGCACAAAGTAATGTTTTATTGTTGTGTTAAATTATGCTTTGTATTGGATGC
SNP_IGA_682254	GAAGGTGACCAAGTTCATGCTGAAGTGGAATTCAGCAGTCTTTCG	GAAGGTCGGAGTCAACGGATTGAAGTGGAATTCAGCAGTCTTTCA	GACTTGCTCTCTTTAGAAGATGAACTGAA	CAGAACATCCTTCACATTTTGACCAGCAGGCCATCATGAAGCCTAGGATCAGCTTCTCCGACTTGCTCTCTTTAGAAGATGAACTGAACAATGCGGATTT[C/T]GAAAGACTGCTGAATTCCACTTCAAGTGGAGAAAACTCACCGAGCTCGCCATTGATGTGTTCATGGCCAGGCAGCAGTCCAATGCAACAACGTCGTTACT
SNP_IGA_691624	GAAGGTGACCAAGTTCATGCTCCAGAACACCCCAAAACCCAGA	GAAGGTCGGAGTCAACGGATTCAGAACACCCCAAAACCCAGC	TAATGCAGGATTTTTCTGCATATGACGAAT	GACTGTGAGCTGACGCCTCCAAGCATATCATCTTGCAGAAACTCACCATCCTCATCAAGAATGGCTTTAGCTTTGCGAGCCAGAACACCCCAAAACCCAG[A/C]TTTTGTGGCCGATTCGTCATATGCAGAAAAATCCTGCATTATTTATAAGGCATTATCAAAATCATCTCTTACCACACAAATTAAGAACAGGGTCACAAAC
SNP_IGA_696341	GAAGGTGACCAAGTTCATGCTTCCTTCTCCATTTCAAAACACCCG	GAAGGTCGGAGTCAACGGATTTTCCTTCTCCATTTCAAAACACCCA	CTTTAAGTGCTTGATGGGCAATAACAGTA	GGAGATTGCCAGATGATAGAAATGTGAGGTGGAGCAAATATCTGTGCAGGAACTTTAAGTGCTTGATGGGCAATAACAGTAGGGGTTATTCTAAGTGTGT[C/T]GGGTGTTTTGAAATGGAGAAGGAAAAACTTAAATGGGTTACAAACAGCTCCCTTCCCATTGATTTTCTGATCAATGATGTGTTGGCTATTAAGCTGGGGG
SNP_IGA_700653	GAAGGTGACCAAGTTCATGCTATTCAGGAACAGAGGACAAGGCT	GAAGGTCGGAGTCAACGGATTCAGGAACAGAGGACAAGGCG	TATCTCCATCTAATTTACCAGTGGGGAAA	TAATGATGTATGAGTGGACATTCACAATCTTCTTTATATTATTTGGAGGTTATCTCCATCTAATTTACCAGTGGGGAAAGGGAACAGCAGTGCAAGCAGG[A/C]GCCTTGTCCTCTGTTCCTGAATCAGTTGATTATGATTCTGGGAAAGGTTAGTTGGCTTGAGTTAATCCAATGCTGATGTACTAAATTTCATCGAATTAGT
SNP_IGA_726222	GAAGGTGACCAAGTTCATGCTAATGACCCTGGAAATGATATAATGGTAATA	GAAGGTCGGAGTCAACGGATTGACCCTGGAAATGATATAATGGTAATG	GGGACTCTTGCATTTAGTATACATGTCAA	CTGCTGGAGCACTGATTCATGGAAAGGAAACTCATTGTTATGCAATAAAATGGATCCTGAACCTAGACAGAAATGACCCTGGAAATGATATAATGGTAAT[A/G]AACGGTCTGATTGACATGTATACTAAATGCAAGAGTCCCAAAGTTGCACGAATGATGTTTGATTCTGTTGCACCAAAGAAAAGGAATGTGGTGACTTGGA
SNP_IGA_717591	GAAGGTGACCAAGTTCATGCTGGATTTGCATCAAATGGTGTGAGC	GAAGGTCGGAGTCAACGGATTCTGGATTTGCATCAAATGGTGTGAGT	TCCCCAACTTTACTATAGCAGTTGCAAA	AGCTTCTTTTGAAGAGAGGTATCTTGCAGATCGACCAGGAGCTTGCTTCAGATAGATCTACGACTGGTATTGTTTCTGGATTTGCATCAAATGGTGTGAG[C/T]TTCAGTCAGAGCTTTGCAACTGCTATAGTAAAGTTGGGGAGTCTCCAGGTTCTTGTCGGAAATGCCGGAGAAATTAGGAAAAAATTGTAGAGTTTTTAATC

SNP_IGA_704075	GAAGGTGACCAAGTTCATGCTGGGTGGGAGACAGTGCATAGAT	GAAGGTCGGAGTCAACGGATTGGTGGGAGACAGTGCATAGAG	GGGTCTGTTGGGTTTAGCTTGGATT	GCTTCTAATCCTTTGCTTTTTAATGGGAAAAAGTGGGCGAATGGGTCTGTTGGGTTTAGCTTGGATTCGTCAATTGTGTAATAATGCTTTTTTGGTAGAA[A/C]TCTATGCACTGTCTCCCACCCATCATCAGCCGTGTCTCTCCTCCCACCTAAACCACCACCACTCCACCCAAATCAATACTATAGCTACCACCATTTCAAG
SNP_Pp07-5647370	GAAGGTGACCAAGTTCATGCTAAGAAGATGAAGAAGGCGCGGTA	GAAGGTCGGAGTCAACGGATTAGAAGATGAAGAAGGCGCGGTG	CCGTCTGTGATGAAATCAACCAGCT	TAGCTGGCCCGTCGGCGCTGATATCGAGCTCCACGGCCACGGCCCTGGAGCTTCCAGGCCCATTCACAGAAGAAGATGAAGAAGATGAAGAAGGCGCGGT[A/G]ACGGGGAGCTGGTTGATTTCATCACAGACGGATTGAAGGCGGTCGATGCGGCGAGCCGCGGCGACGATCCTGCATCCAGCTTTAGCCAAGTCCAAGCATA
SNP_IGA_752104	GAAGGTGACCAAGTTCATGCTGCCTTACTTGAGTACCTGAAAGC	GAAGGTCGGAGTCAACGGATTAGGCCTTACTTGAGTACCTGAAAGT	GAAGTACGTAGTCCTTTTAGTACCCGT	CGTTGATGGGTGTTCGACGCTGGAATTCATATACAAATATGAAAAGTTAGCCTCAATTCTGGTACCGCTCCATGTGAGGCCTTACTTGAGTACCTGAAAG[C/T]GCACGGGTACTAAAAGGACTACGTACTTCAGCCTTTCAGGTACTAAAAATACATTTGCTGCTTTGCAATTTGCAATTTGTATGCTGCATGGTTCTGTACG
SNP_IGA_758767	GAAGGTGACCAAGTTCATGCTGGTTGAACTGGCAGCAAACAATGAT	GAAGGTCGGAGTCAACGGATTGTTGAACTGGCAGCAAACAATGAG	TTCGGATGGCGTCACGTTGTAGAA	CTTTATCATGTTGAGCGAATGCCTTTTTAAAATGATCTCCAGTTTGCTCTCGTACTTCGGATGGCGTCACGTTGTAGAACACGGGCAAAATCAATCTGGA[A/C]TCATTGTTTGCTGCCAGTTCAACCATTTTCGCAAGTTCATCCAGACACCATGTGGAGGTCGCATAGTTTGTTGAAAGAATGATGACTGAAATTTTCGATT
SNP_IGA_774557	GAAGGTGACCAAGTTCATGCTGCACTATCAGGATCTGAGAAGATA	GAAGGTCGGAGTCAACGGATTGCACTATCAGGATCTGAGAAGATG	CTCTGCTGATGGTTTCGAGACTACAA	AGCACAATAGTAAAACTATGGCCACAATCAAGCACTTTTACAATCGGGGACAACAAAACTGAAAACAGAGTTAGCCTGCACTATCAGGATCTGAGAAGAT[A/G]TTAACATTCCCACACTTTGTAGTCTCGAAACCATCAGCAGAGGTTTGTTTAAGACTCAAAGGAACCTTCAGCTTGCAGTTGATGTTCCGTGACGGCGTGT
SNP_IGA_776826	GAAGGTGACCAAGTTCATGCTGCCTCTGTAGCTTCCACCAAAT	GAAGGTCGGAGTCAACGGATTGCCTCTGTAGCTTCCACCAAAC	TTTGCTGATGATATTGCAGGCGACATTT	TCTACACTAGAAATTCTGGTATTCTTGATCACTAGAGGACATGGGAGAGCTCTTCTTTTGTTTGCTGATGATATTGCAGGCGACATTTCCATAGGGAAGC[A/G]TTTGGTGGAAGCTACAGAGGCACGCGGCAGAGGGGAAAAGAGATTCGGAGAAAGATGAGCAGAAGCAGTAACAATAGCCATTTGATAGATATCAGAGTAG
SNP_IGA_781003	GAAGGTGACCAAGTTCATGCTGCACTTGTGAACCATCAGCCAA	GAAGGTCGGAGTCAACGGATTGCACTTGTGAACCATCAGCCAG	AACTGAATGGACATTGTGGACATGTCTTA	GTAAATTTAAATTATATAAGCAAGGGAAGAACCTTGAATGTTTTATAAGGGGTTGCATTTGATAAAACCATTGAAGTGTGCACTTGTGAACCATCAGCCA[A/G]CAAAACCTAGAATAAGACATGTCCACAATGTCCATTCAGTTAGATTTAAGTTTCAAAACTGCCTCATTTAGGAAAGCAAGAAGAAACCAACCCCGTTGAC

SNP_IGA_784777	GAAGGTGACCAAGTTCATGCTGTTATATACATCAAGAGAGGCACATACAA	GAAGGTCGGAGTCAACGGATTATATACATCAAGAGAGGCACATACAG	GGTTCCATTTTTTCTTCTTAATCTCCACAT	GATGGCACTGGAAATTTCACAAATGTGACGGATGCCGTGTTAGCAGCACCAGATTATAGCATGAGAAGATATGTTATATACATCAAGAGAGGCACATACA[A/G]AGAGAATGTGGAGATTAAGAAGAAAAAATGGAACCTAATGATGATTGGAGATGGTATGGATGCTACTATAATCTCTGGTAACAGAAGCTTTGTGGATGGC
SNP_IGA_794167	GAAGGTGACCAAGTTCATGCTGAATATGCAGTTGGACACTCTGACT	GAAGGTCGGAGTCAACGGATTAATATGCAGTTGGACACTCTGACC	GAACCTCTTTGATAGGATCAGGCACTT	AGTATTTTAAGAACGGTGTCCCCAGGCATCCTGTTAACTTGTAGATGGCGAACCTCTTTGATAGGATCAGGCACTTCACTTTTCATTATTTCATTGTTCA[A/G]GTCAGAGTGTCCAACTGCATATTCAGGTTCCAATTCTTTAACAATATTATGATAAAATTCATCAACCTCAGGGGAGTCTGGGTTGGAGGACGTAGGTTTC
SNP_IGA_797492	GAAGGTGACCAAGTTCATGCTAGGCCTCGTAACCAATCATTACTC	GAAGGTCGGAGTCAACGGATTATAGGCCTCGTAACCAATCATTACTA	GAGTTTGAAGATCGTTGGAAAGAGATGAT	AATCTATTAAATTTGCCTTGGAGAACATTGTGTATGATTCATTGACCAATATTGAGTTTGAAGATCGTTGGAAAGAGATGATTGAGAAGTATGAGTTACA[G/T]AGTAATGATTGGTTACGAGGCCTATATGATGAAAGACGTCGTTGGGTGCCAAGCTTTGTGAAAGGAAGTTTTTGGGCGGGCATGTCTACCACACAACGAA
SNP_IGA_803699	GAAGGTGACCAAGTTCATGCTCACCACCTCCACTCTGATCTTGT	GAAGGTCGGAGTCAACGGATTACCACCTCCACTCTGATCTTGC	GGAAGGAAGGGAATCACAGATTCGAA	GTGAGTCTCATTATACAGTCCTTTTTTCCCTAGATACCACTGTTCAAGATGAGAATGAACTGGAAGGAAGGGAATCACAGATTCGAAAAGCTTTTGATGC[A/G]CAAGATCAGAGTGGAGGTGGTGGTTTTATTAGTGTGGAAGGCTTTCATCAAGTCCTTAAAGAAACTAATGTAGAACTTCCAACTGAGAAGGTTGACCTCC
SNP_Pp08-3702593	GAAGGTGACCAAGTTCATGCTGGGATGTGCCGAGGCACG	GAAGGTCGGAGTCAACGGATTGTGGGATGTGCCGAGGCACA	GCAAGCTTTGTCTCGCCTTAAGCAT	CAACATCCCTTTTGCCTGCAGCACCCCCGTGCGTAGCCACATTGCCTTGTAACATGCAAGCTTTGTCTCGCCTTAAGCATCCCTTCACCTTCGGCACCCC[C/T]GTGCCTCGGCACATCCCACTGCGACATGCAAGCTTTGTGCATTTGACACCCCCGTTGCCTTCAGCACCCCCATGCCTAGCCACATTGCCTTACAACATGC
SNP_IGA_821894	GAAGGTGACCAAGTTCATGCTTTTGAATGATTTCATTCACACCTCCC	GAAGGTCGGAGTCAACGGATTCATTTTGAATGATTTCATTCACACCTCCT	CATTATCATCTGGTTCGTTGGCAGGAT	TATACAAGTGTCCAGGCTCTCCGTTGATTGTGAAGGCATCTGAAACGTTTGGATCGCCACCTGTTGAGAGAGCATTTTGAATGATTTCATTCACACCTCC[C/T]TTGTACCAGGATCCTGCCAACGAACCAGATGATAATGAAAAATAATTATATGGGATTCGATGTTGCAACTGTATGATGTAACATTTTTTGTTTTCATGTC
SNP_IGA_851849	GAAGGTGACCAAGTTCATGCTTCAATTGTTTGGGAGAACTTTATTAAG	GAAGGTCGGAGTCAACGGATTCCTTCAATTGTTTGGGAGAACTTTATTAAA	GCTACCATATGCATACACTTTATTGCAATA	GACTCAACAAGTTTGATGTCCCATTTTTTTTGCTACCATATGCATACACTTTATTGCAATATTTGCATTTTGCCTTAGGATTTTTTGGATCATCTTGCAT[C/T]TTAATAAAGTTCTCCCAAACAATTGAAGGAGGTCTAGCAGGTTTTCTCTAACCATCTTCAATGGGAGCAGAATCATTGGCTTTGGTTTCTTGTGGCAACA

SNP_IGA_860815	GAAGGTGACCAAGTTCATGCTTATTGTGCTACTTTTAGGTCAAACTTTCA	GAAGGTCGGAGTCAACGGATTGTGCTACTTTTAGGTCAAACTTTCG	GTTCTGCTGTCTTGCCATCAGCTTT	ATTACTGTCCAGCTCTTGTCAGAGATTGATACCTTTTCTATTACTAATCTGTCATTTTGTCTGATGTATTTTTATTGTGCTACTTTTAGGTCAAACTTTC[A/G]GAGAAAGCATTGTTGCTGGAAAAAGCTGATGGCAAGACAGCAGAACTTACAGCAAAGGTTAATGAACAACAAAAGTTAATCCAAAAGTTGGAGGATGACA
SNP_Pp08-13670362	GAAGGTGACCAAGTTCATGCTGGGCTGCTTACAGGTGTTTTGTG	GAAGGTCGGAGTCAACGGATTAGGGCTGCTTACAGGTGTTTTGTA	ATGCTAAGCCAAAATCTTATCTGAAAGCAA	TAAGAAGCCTTATTTTCCCCTTTTGATGCTCAACTCATGCTGACCCTGCTCCCATACCACAAGCTCTTCATGCTAAGCCAAAATCTTATCTGAAAGCAAG[C/T]ACAAAACACCTGTAAGCAGCCCTCATTTTCATTGGTGAAGGTAACCAGAACACTTCCTAAGGTTTTGCTCCCCTTTCGAAGTGCTTTTGGGGCTTGATTT
SNP_IGA_871082	GAAGGTGACCAAGTTCATGCTGTACAAGAAAGCAAACCCCAGCAA	GAAGGTCGGAGTCAACGGATTACAAGAAAGCAAACCCCAGCAG	CCAATCTTTGGAGATGGTTTACCAGAATA	TTTTCAGAGCTCTTCAAACAGTTCCCATTCTGATCAGTCTTCACTAAAGCTCTGGCTTTCTTCTTCCCTCTTAGTCTGTACAAGAAAGCAAACCCCAGCA[A/G]TGAAGCTATGACTGCACCAAAAATATTCTGGTAAACCATCTCCAAAGATTGGTGCAAAAAGGGTTAAAGAAGATTATGGTGGCACTCTCTGAATTTAAGT
SNP_IGA_878044	GAAGGTGACCAAGTTCATGCTCAGTTGCAAGGGAGAACAGGGTA	GAAGGTCGGAGTCAACGGATTAGTTGCAAGGGAGAACAGGGTG	GGTTGTATACTCTGCTCACCGGAA	ACATCAAAGTTTGTTACTTTTGGATCAAATTATTTCTTGAAGTTACTGTAGGTAAAATTGGATGGGAATTTACCTGGACAGTTGCAAGGGAGAACAGGGT[A/G]ATTCCGATTTTCCGGTGAGCAGAGTATACAACCCCTTCGGACTCACTTCCGAGCTTAATTCCGGTAGCCCAGCCGGCGACCCCAATGGCATAACCAGATA
SNP_IGA_883524	GAAGGTGACCAAGTTCATGCTAGAGGTCATTATACTGTGAAGGTGC	GAAGGTCGGAGTCAACGGATTGAGAGGTCATTATACTGTGAAGGTGA	CGCTTTCTCAAGGTTTTCCCATCTCTT	AACATTCTGTTTTCTCATTAATGTAGCCGCCCGATACATGTAAGGGTAAGTGAGAGTTGGGTCCAGCTCAGACGCTTTCTCAAGGTTTTCCCATCTCTTA[G/T]CACCTTCACAGTATAATGACCTCTCCTGATACATCCATCCAAGTGGGGTAAGAGAGCAGATTACAGAGCTCATCTTTTCATACGACCAAAGTTTATGGCC
SNP_IGA_884755	GAAGGTGACCAAGTTCATGCTGTAGAAGGCAAGGAAGATATGGT	GAAGGTCGGAGTCAACGGATTGTAGAAGGCAAGGAAGATATGGC	TCACCTGCAACAGGTTGCTCATCAA	AGCCACCATCCCTTCAGCAGATGGAAGGCATCATGCTTCCACCAGTAGTCCATCTGCTTCACCTGCAACAGGTTGCTCATCAACAGTACCTGAAAAAGGT[A/G]CCATATCTTCCTTGCCTTCTACAGATACCAAGTTTTCCCAGAAATCTGGGTTGGAAGAGATATCTGGACCTAAGACCCCAGTTTGCACCTCCGAATCCCC

Table S3. Heterozygous SNPs for 'Sweet Dream' and SNP density per chromosome

Chromosome	#Heterozygous SNPs	Total chrom size (bp)[1]	SNPs/Mb
Pp01	6,913	47,851,208	144.5
Pp02	22,078	30,405,870	726.1
Pp03	12,325	27,368,013	450.3
Pp04	14,278	25,843,236	552.5
Pp05	9,937	18,496,696	539.7
Pp06	10,554	30,767,194	343.0
Pp07	11,701	22,388,614	522.7
Pp08	6,665	22,573,980	295.3
Total	94,451	225,694,811	418.7

[1]Source:www.rosaceae.org

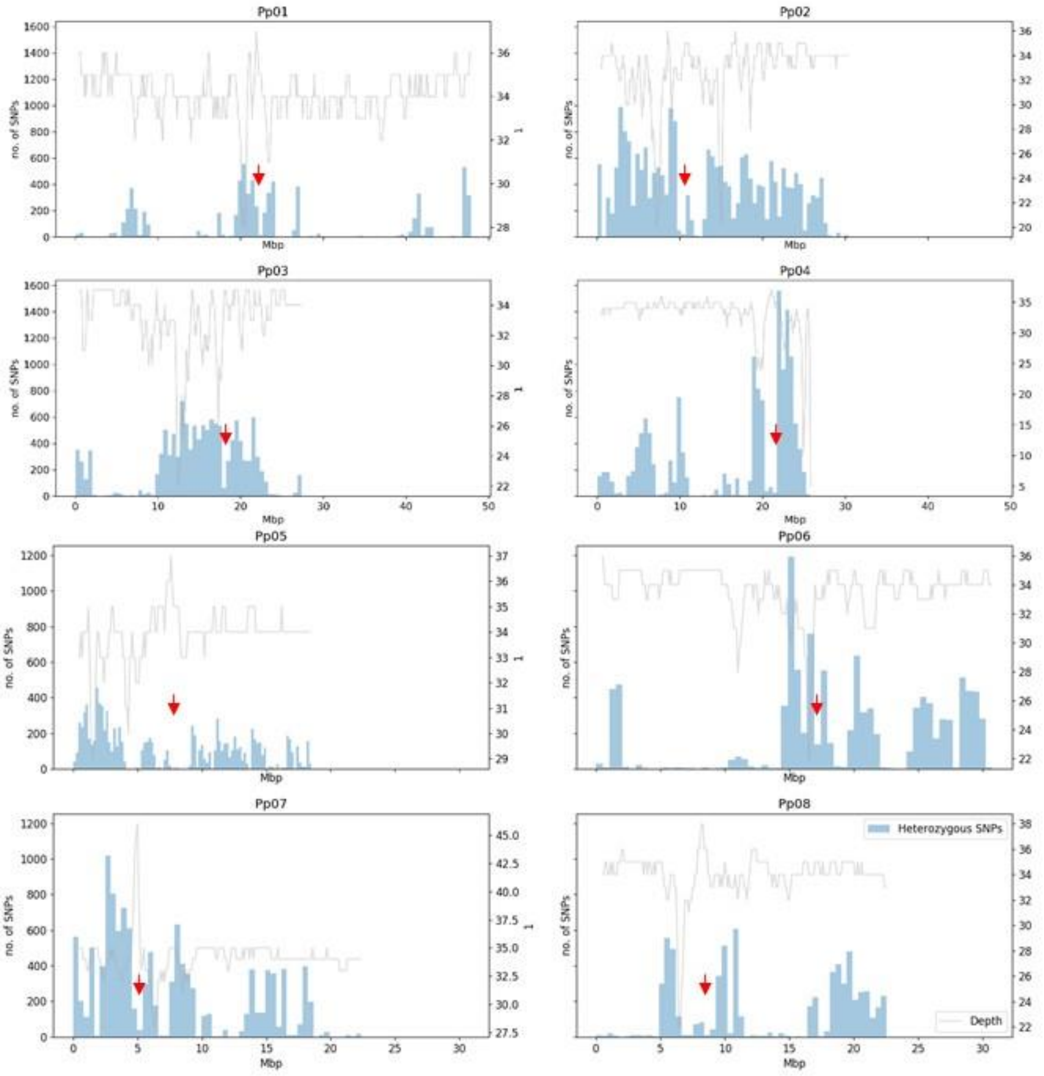

Figure S1. SNP and depth distribution in ’Sweet Dream’. The distribution of heterozygous SNPs is shown across the eight chromosomes of the peach genome in bins of 500kbp. Grey line represents median read coverage across chromosome, calculated in windows of 500Kb with a step of 100kb. Red arrows indicate the approximate positions of the centromeres.

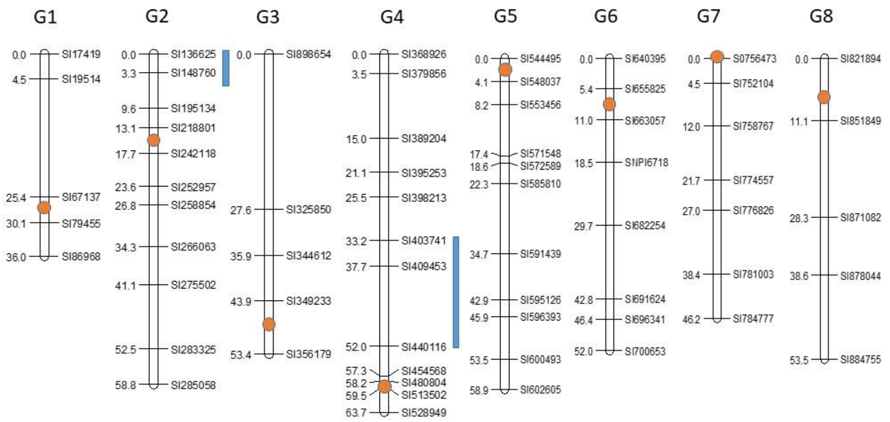

Figure S2. Linkage map of peach ‘Sweet Dream’ with 64 SNPs. Red dots indicate the approximate positions of the centromere. Blue bars to the right of linkage groups 2 and 4 are the positions of the major gene/QTL hotspots in the cherry and peach genomes (Aranzana et al. 2019).
